# Treatment of thromboangiitis obliterans (Buerger's disease) with bosentan

**DOI:** 10.1186/1471-2261-12-5

**Published:** 2012-02-14

**Authors:** Joaquin De Haro, Francisco Acin, Silvia Bleda, Cesar Varela, Leticia Esparza

**Affiliations:** 1 Department of Angiology and Vascular Surgery, Hospital Universitario de Getafe, Madrid, Spain

## Abstract

**Background:**

This study assessed the effectiveness and safety of bosentan when administered to thromboangiitis obliterans (Buerger's disease) patients.

**Methods:**

A clinical pilot study was designed in which patients with ulcer and/or pain at rest were treated with bosentan p.o. at a dose of 62.5 mg twice daily during the first month, which was thereafter up-titrated to 125 mg twice daily. The study endpoints were clinical improvement rate, major or minor amputation rate, haemodynamic changes, changes in endothelial function and angiographic changes.

**Results:**

Seven out of 12 patients were male (58%). Median age was 39 years (range 29-49). The median follow-up was 20 months (range 11-40). All patients were smokers. With bosentan treatment, new ischaemic lesions were observed in only one patient. Overall, clinical improvement was observed in 12 of the 13 extremities (92%). Only two out of 13 extremities underwent amputation (one major and one minor) after bosentan treatment. After being assessed by digital arteriography with subtraction or angio-magnetic resonance imaging, an increase of distal flow was observed in 10 out of the 12 patients. All patients experienced a statistically significant improvement in their BAFMD values (mean: 1.8 at baseline; 6.6 at the end of the treatment; 12.7 three months after the end of the treatment; p < 0.01).

**Conclusion:**

Bosentan treatment may result in an improvement of clinical, angiographic and endothelial function outcomes. Bosentan should be investigated further in the management of TAO patients. Larger studies are required to confirm these results.

**Trial Registration:**

ClinicalTrials.gov: NCT01447550

## Background

Buerger's disease, also known as thromboangiitis obliterans (TAO), is a thrombotic, occlusive, non-atherosclerotic, segmental vasculitis of small and medium-sized arteries and veins, which may involve both upper and lower extremities. The onset usually occurs in people of around the age of 45, and is more frequent in male smokers. As a consequence of the increase in tobacco use, an increase in the incidence of TAO in women has been observed in the last 20 years [1,2].

Intermittent claudication and, in more advanced cases, pain at rest are the predominant clinical symptoms. Distal ischaemic lesions (trophic and ulcerations) are frequently observed by means of physical examination. Clinical course is characterised by alternating periods of exacerbation with periods of remission. Angiographic studies reveal a distal and segmental involvement of the vasculature of the extremities. Recanalisation is frequently demonstrated, showing a typical image (corkscrew collateral vessels) [3]. Skin disorders such as migrating phlebitis or Raynaud-like colour changes may be associated with TAO.

Giving up smoking is the most important therapeutic measure in TAO patients [4]. In fact, it leads to dramatic improvement of the symptoms and lesions. Amongst the drugs used to manage TAO, prostacyclin (PGI2) or its analogues (iloprost, beraprost, trepostinil sodium), aspirin or streptokinase (as a thrombolytic) are the most important. Revascularisation by means of a by-pass surgery or endovascular procedure is usually not possible as a consequence of the predominantly diffuse and distal location of the lesions in the veins and arteries involved. Endothelin-1 (ET-1) is a potent vasoconstrictor peptide, which exerts its action by targeting two transmembrane receptors (ETA and ETB). Pharmacologic ET-1 receptor blockade, may be single (ETA or ETB) or dual (both, ETA and ETB) [5]. Bosentan is a dual ET-1 receptor antagonist, administered orally, which is approved in the European Union to treat pulmonary arterial hypertension in systemic sclerosis patients and to prevent the occurrence of new digital ulcers in systemic sclerosis patients with ongoing digital ulcers. The results of a small retrospective study [6-8] and a prospective study [9] suggested that bosentan could have a role in healing ongoing digital ulcers in systemic sclerosis.

Based on these previously reported studies on digital ulcers in Systemic Sclerosis patients, we designed a pilot study in order to assess both the safety and effectiveness of bosentan in the pharmacologic management of ischaemic symptoms and ulcers in TAO patients, the results of which are reported herein.

## Methods

### Patients

In order to be included in the study, the patients had to have a previous diagnosis of TAO, fulfilling the Shionoya criteria [10]: 1) onset of distal ischaemic symptoms in one extremity before the age of 50 years, 2) smoker or history of smoking, 3) distal ischaemia of the extremity confirmed by a non-invasive test (ankle-brachial index [ABI], toe-brachial index or partial pressures), 4) absence of thrombophilia, autoimmune disease, diabetes, hyperlipidaemia, trauma or a proximal source of underlying embolism, 5) arterial tree proximal to the distal segment of the healthy superficial femoral artery, and 6) occlusive distal disease with specific elements or actual pathological findings demonstrated angiographically or by means of angio-magnetic resonance imaging. The consecutive patients included in this study were required to meet the following inclusion criteria: critical ischaemia in any extremity, pain at rest or non-healing ischaemic ulcers, present for at least four weeks with no evidence of improvement in response to conventional treatment; not being candidates for surgical or endovascular revascularisation of the extremity studied; objective confirmation of the ischaemia in the affected extremity by means of an ABI at rest of less than 0.6, if it was the lower extremity, or a partial pressure of less than 50 mmHg in the upper extremity, in two consecutive assessments at least one week apart. In each patient included with pathological conditions in the upper extremities was performed a study to assess blood flow in the upper limb affection performing an Allen test, the determination of the Digital/Brachial Index (DBI), Radial/Brachial Index (RBI) or Ulnar/Brachial Index (UBI) and the determination of the partial pressure in the radial artery. DBI, RBI and UBI indexes were defined as the ratio of the segmental arterial pressures of the digital arteries, radial and ulnar, respectively, and the partial pressure in the brachial artery.

We considered a ratio < 0.7 in DBI, RBI and UBI as an indicator of an inadequate blood perfusion in the upper limb [11-13]. Conventional treatment includes: advice for patients on giving up smoking; surgical or endovascular revascularisation of the extremity when possible, peripheral sympathectomy; prostanoid vasodilator iloprost (in a 21-day cycle). The exclusion criteria were as follows: any known contraindication for treatment with bosentan such as patients with liver failure or liver diseases, with anaemia or pregnant women; presence of arteriosclerotic risk factors, apart from smoking (hypertension, hyperlipidaemia and/or diabetes mellitus), any other medical or psychological condition which may represent, in the investigator's opinion, a contraindication to either bosentan treatment or to adequately performing the treatment and procedures of the study. Patients with other causes or forms of peripheral vasculopathy such as popliteal entrapment syndrome, cystic adventitial disease, collagenopathy and hypercoagulability (e.g. the presence of Factor V Leiden or antiphospholipid syndrome) were also excluded from the study.

### Design

The study was designed as a pilot, open-label, non-controlled, non-randomised, single centre clinical study, where patients previously diagnosed with TAO received treatment with bosentan in a compassionate use programme. No concomitant medication was administered during the study period except analgesics and antibiotics.

The study protocol was approved by the Institutional Review Board of the participating centre and regulatory authorities. Patients were required to give written informed consent before enrolment.

The study endpoints were clinical improvement rate (absence of new trophic lesions, ulcer healing process, pain relief, complete absence of pain), major or minor amputation rate, haemodynamic changes as measured by means of ABI, changes in endothelial function as measured by means of the brachial artery flow-mediated dilation test (BAFMD), and angiographic changes as measured by an arteriography with digital subtraction or angio-magnetic resonance imaging (MRI).

Clinical therapeutic success was defined as the complete healing of the distal ischaemic trophic lesion or complete ischaemic pain relief at rest if the patient presented no lesion.

### Treatment

All included patients received a treatment regimen with bosentan for compassionate use, after strict compliance with the inclusion criteria and the absence of exclusion criteria were ascertained. Bosentan therapy consisted of a month's treatment with 62.5 mg twice daily (bid) administered orally. The initial dose was doubled to 125 mg bid after the first month if significant adverse events attributable to bosentan were ruled out. This full-dose regimen (125 mg/12 h) was maintained for the following three months or until total healing of the ulcers, provided that the liver function tests and blood cell count remained within the normal range. Patients were given analgesic treatment, as necessary, to control pain at rest. The ischaemic lesions and necrotic ulcers were treated with standard daily care and antibiotherapy as necessary. Special methods of treatment were not used and nor was absolute bed rest prescribed during the study period.

### Patient assessments

All patients were monitored fortnightly on an outpatient basis, at baseline, weekly for the first eight weeks after the start of bosentan treatment, and monthly thereafter. Baseline assessments included medical history, concomitant medication requirements, (mainly analgesics or antibiotics), and a complete physical examination. Laboratory assessments, such as liver function tests and blood cell counts were performed, as well as frequent immunological tests.

In addition, a pregnancy test was performed in women of childbearing age. The ischaemic ulcers were documented with colour photographs for all patients before the BAFMD was assessed. The target extremity for assessment purposes was defined as the one presenting the trophic lesion or causing pain at rest.

Baseline haemodynamic studies included the ABI at rest, and the partial brachial pressures (absolute value).

Bioactivity of bosentan was assessed by means of BAFMD, as an indirect measurement of the endothelial function that is based on the capacity of nitric oxide (NO) production released by the endothelium, which may lead to arterial dilation. This measurement is achieved by means of an ultrasonography, a non-invasive technique based on parietal stress, caused by increased flow after a period of ischaemia. This causes the opening of ionic channels, thereby increasing the concentration of intracellular calcium and stimulating the enzymatic activity of endothelial NO synthase. As a result, there is a relaxation of smooth muscle, together with dilation of the brachial artery [14].

Measurement of BAFMD: Before the test, all patients fasted for a minimum of 12 hours and were advised not to partake in physical exercise or smoke. They were placed in dorsal decubitus, in a calm environment and under controlled room temperature. A Doppler ultrasound machine (HD11 XE, PHILIPS, Eindhoven, The Netherlands) was used with a L12-3-MHz linear probe. After obtaining an image at rest, ischaemia was applied at a pressure of 250 mmHg over a period of five minutes. Another image was obtained 60 seconds after releasing ischaemia. The BAFMD was calculated as the as the post-ischaemic diameter after 60 seconds, minus the diameter at rest, divided by the diameter at rest and expressed as a percentage.

BAFMD was assessed according to a previously described technique [14], in each patient before and after treatment with bosentan and three months after the end of treatment. An arteriography with digital subtraction (or an angio-MRI) was performed at baseline and three months after the last dose of bosentan.

During follow-up, all the information on the clinical symptoms, evolution of trophic lesions, presence/absence of major or minor amputation, smoking and patient's physical activity was collected. This information was obtained directly from the guided anamnesis, physical examination and photographs that were taken at the successive follow-up visits.

### Outcome assessments and techniques

The criteria we used to document the changes in the status of the target extremity were taken from the Inter-Society Consensus for the Management of Peripheral Arterial Disease (TASC II) consensus-based standard recommendations [15]. The haemodynamic effect of bosentan on macrocirculation was determined by means of the ABI, which was obtained by taking the systolic pressure figure in the posterior tibial artery at maleolar level or the dorsalis pedis artery and dividing this measurement by the brachial artery systolic pressure. These measurements were always taken with the patient in the supine position and following a rest period of 15 minutes, using a continuous bidirectional Doppler ultrasound device. The drug's bioactivity was shown through the monitoring of the improvement in patient endothelial function, produced by the treatment and indirectly measured by means of BAFMD, as was previously described.

### Statistical analysis

Qualitative variables were described by frequencies and percentages and quantitative variables by mean ± standard error or median and range. Differences between time points were compared, using the Wilcoxon test for dependent samples and the Friedman test for repeated measurements. The threshold of statistical significance was established as p < 0.05. Statistical analyses were performed using SSPS version 16.0.

## Results

### Patients

A total of 12 consecutive patients were included in the study (58% men). One patient had both an ulcer on one foot and ulcers on their fingers. As a consequence, results refer to 12 patients and 13 extremities. The median follow-up was 20 months (range 11-40). Table 1 shows the demographic and clinical data of the patients as well as BAFMD results. Prior to the treatment with bosentan, three extremities in three different patients (25%) had undergone revascularising procedures and three patients (25%) had a lumbar sympathectomy. Ten out of 12 patients (83%) had previously been treated with a 21-day prostaglandin regimen. In all these pre-treated patients, bosentan treatment began after a minimum period of 15 days with no clinical improvement in the symptoms or healing of the ischaemic ulcers. Eleven patients with trophic lesions in one or more finger(s) or toe(s) were included. One patient presented lesions on the back of the foot at baseline. Only one patient presented ischaemic pain at rest with skin integrity at baseline. The upper extremity was involved in three patients, in the form of ulcerous lesions in the pads of the fingers or periungual areas.

**Table 1:** Patient characteristics and results of brachial artery flow-mediated dilation test

**No**.	Age	Gender	Prior treatment	Give up smoking	Months F/u	Pre-treatment clinical results	Post-treatment clinical results	BAFMD Pre-treatment	BAFMD Post-treatment	BAFMD 3 months after
1	35	F	PG + LS	YES	20	Ulcer in toe	Healing	1.1	6.7	11.6
2	36	F	PG	YES	40	Ulcer in toes	Healing	0	5.2	17.1
3	32	M	PG	NO	21	Ulcer in fingers	Healing	1.5	6.9	10.8
4	46	F	PG	NO	20	Ulcer in fingers	Healing	0.8	6.5	12.3
5	47	M	PG	NO	12	Pain at rest	Pain relief Claudicates 300 m	2.3	7.1	12.6
6	46	M	PG	NO	11	Ulcer in toes	Healing	3.5	8.9	9.8
7	29	M	PG	NO	35	Ulcer in toes	Healing	1.2	4.1	9.6
8	39	F	BP (occlude)	NO	31	Ulcer in toe	Healing	0.3	5.9	11.3
9	45	M	BP (occlude)	NO	18	Ulcer in toe	Ulcer Improvement	0.9	4.9	10.1
10	39	F	PG + LS	YES	16	Ulcer in forefoot	ICA	3.7	7.7	16.9
11	22	M	PG + LS	NO	13	Ulcer in toes	Healing. Amp. 3^rd ^toe	4.1	7.8	17.9
12.a	49	M	2 BP (permeable) + PG	YES	12	Ulcer in toe	Healing	2.3	6.9	12.3
12.b	49	M	PG	YES	12	Ulcer in fingers	Ulcer Improvement	2.3	6.9	12.3

Abbreviations: BAFMD: brachial artery flow-mediated dilation test; ICA: Infracondylar amputation; Amp: Amputation; BP: Bypass; F: Female; M: Male; PG: Prostaglandins; LS: Lumbar Sympathectomy; F/u: follow-up.

Seven patients were male (58%). The median age of the patients was 39 years (range 29-49). Seven patients (58%) had previously been diagnosed with at least one episode of phlebitis migrans. All patients were current smokers and had been heavy smokers for years. Eight patients (67%) were unable to completely abstain from smoking during the follow-up.

We discarded the presence of associated autoimmune diseases in patients due to all the immunological testing included, as tests for C-reactive protein, rheumatoid factor, antinuclear antibodies, anti-DNA antibodies, antiribonucleoprotein antibodies, anti-Ro antibodies, anti-Sm antibodies, anti-Jo-1 antibodies and antineutrophil cytoplasmic antibodies were negative. Levels of complement C3 and C4, and thyroid stimulating hormone, were normal in all patients. Tests for anticardiolipin antibodies were negative, as were tests for syphilis, HIV, and hepatitis B and C. Diagnoses of cardiogenic or aortic embolism, early-onset occlusive atherosclerotic disease, collagen disease and hypercoagulable state were excluded.

As shown by angiography, the infrapopliteal arteries were involved in the 10 patients with lesions on the lower extremities, whereas the upper extremities were involved in three patients (25%). Either a digital arteriography with subtraction or an angio-MRI documented the typical findings of Buerger's disease in all 12 patients at baseline. These findings included segmental occlusive disease, mainly affecting the tibial, forearm and digit vessels, as well as the plantar and palmar arches, although this involvement extended proximally to the popliteal artery in two cases. In all cases the typical corkscrew collateral vessels were observed and the proximal vessels to the superficial femoral artery were free of any sign of disease.

### Effectiveness

During the follow-up, no new ischaemic lesions were observed in the target extremities in all but one patient whose ulcer had begun to heal and who, three months after the end of bosentan treatment, had a relapse of an ulcer on their toe. This relapse was treated with a new four-month course of bosentan which managed to heal the lesion. Only one patient required prolonging the administration of bosentan beyond four months in order to achieve total healing of the ulcers.

Overall, clinical improvement was observed in 12 of the 13 extremities (92%) treated, while only one extremity which presented lesions at the level of the forefoot ultimately required major amputation below the knee. Of the 12 extremities that improved, ten (77%) achieved complete clinical therapeutic success (healing or complete pain relief) [Table 1]. Moreover, a minor amputation of one toe was performed with conservation of the extremity and healing of the ulcers of the other toes in one patient. Two out of 13 extremities (15%) underwent amputation (one major and one minor) after bosentan treatment. Thus, an extremity conservation rate of 92% was achieved.

All 10 of the lower extremities treated presented an ABI lower than 0.6 before treatment. After the end of bosentan treatment, the ABI increased by more than 0.1 in two patients. These differences were statistically non-significant when compared with baseline values. None of the three target upper extremities, which presented a partial pressure below 50 mmHg at baseline, experienced an increase in partial pressure above 10 mmHg after bosentan treatment.

**Figure 1:**
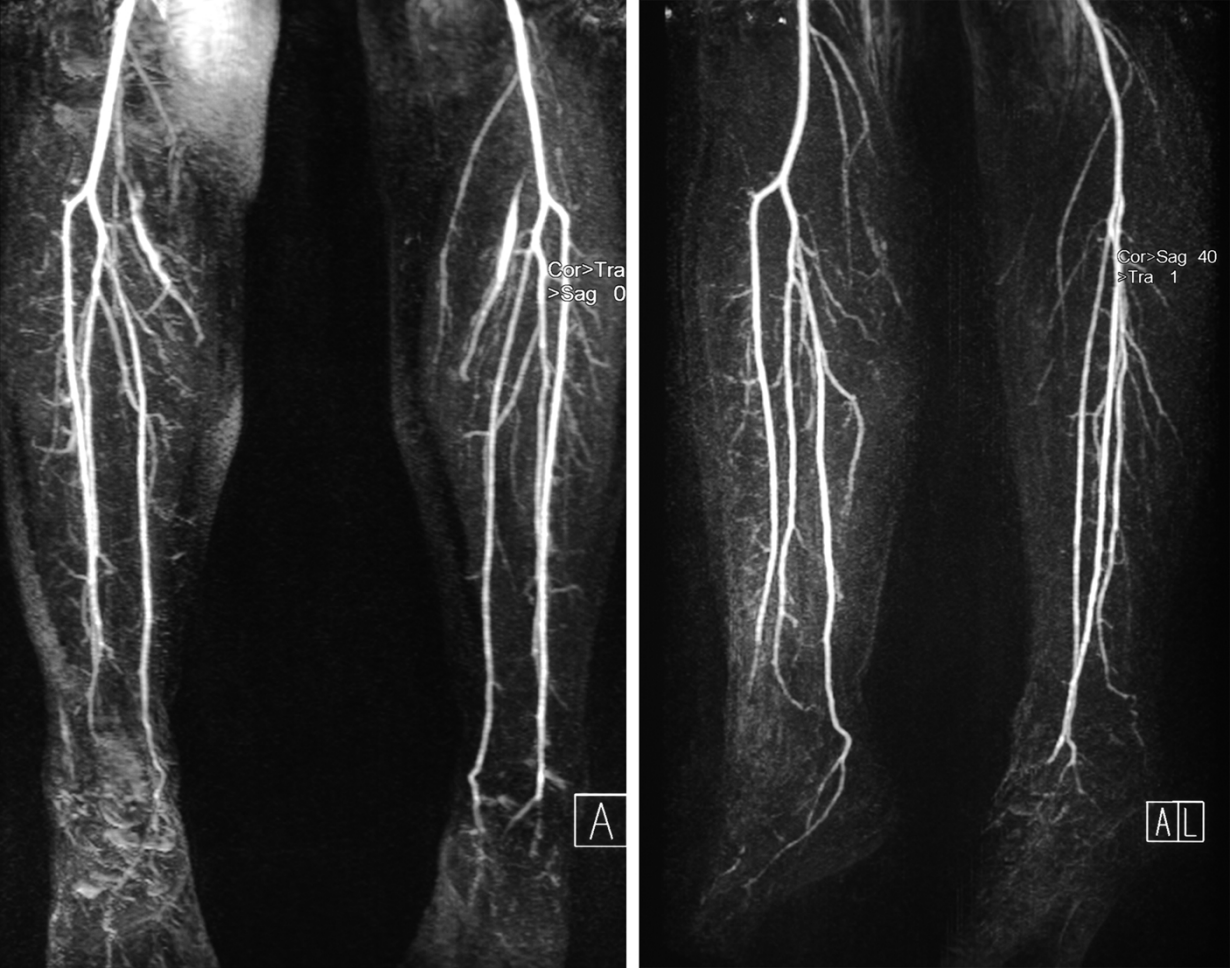
**Increased intensity of the signal in the affected vessels and visible collateral vessels**.

As assessed by digital arteriography with subtraction or angio-MRI, after treatment with bosentan, evidence of a qualitative improvement in distal flow was observed in 10 out of the 12 patients, when compared with baseline This result included an increase in the intensity of the signal in the affected vessels at baseline angiography as well as an increase in the number of visible collateral vessels in two patients (Figure 1).

The BAFMD test was well tolerated in all patients. Baseline endothelial function was reduced in all patients (mean BAFMD = 1.8 ± 1.3%). After the end of bosentan treatment, all patients experienced a significant improvement in their values at this endpoint (mean BAFMD = 6.5 ± 1.3%), making this difference statistically significant (p = 0.002). Three months after the end of treatment with bosentan, the BAFMD values were also higher than they were at the end of the treatment, reaching a mean value of 12.7 ± 2.9% (p < 0.001).

### Safety

No patient suffered serious adverse effects due to treatment with bosentan leading to withdrawal of the treatment. The repeated liver enzyme measurements remained within the range of normality. Moreover, blood cell counts were normal in all but one patient who presented haemoglobin values in the lower limit and was treated with enteral iron. We did not find statistical differences in the levels of blood pressure in our patients after the administration of Bosentan. The only documented bosentan-associated adverse event was the occurrence of transient oedema in the lower extremities in 3 patients (25%), consistent with the vasodilatory effect of the distal bed associated with bosentan use. No new safety signals were observed.

## Discussion

As TAO is associated with both distal ulcers in the extremities and the possibility of amputation, it frequently involves social problems and a worsening in the quality of life of the affected patients [16]. Only a few pharmacological and surgical options (of controversial efficacy) are available to date [17]. New therapeutic options with a higher efficacy than the current ones are clearly needed in order to properly manage patients affected by TAO.

Our study is a small pilot study and, as a result, it is not possible to draw firm conclusions from it. On the contrary, its objective should be to generate hypotheses which firstly require further confirmation in larger prospective studies, and secondly, a definitive assessment in comparative randomised trials. In any case, the characteristics of this disease, the low incidence and the lack of effective treatments that improve the course of the disease or correct the cause, contributes to serious ethical difficulties in carrying out this type of studies in this particular disease. We consider special and important the value of the results obtained in our study and the immediate applicability in the clinical practice. This study should be considered in this setting. However, our results show that bosentan therapy was associated with several clinical and endothelial function-related outcomes in patients with TAO, which may be promising. This report adds data to previous single reports [18,19] on the efficacy of bosentan and the management of patients with TAO.

Both clinical improvement and complete healing of the ulcers were achieved in the majority of the target extremities despite the documented failure to give up smoking in the majority of the patients. Moreover, the only major amputation was performed in a patient who gave up smoking definitively. This result compares favourably with a 19% major amputation rate in 69 patients who continued smoking, previously reported in a series involving 110 patients with TAO [16]. The prostacyclin analogue iloprost has shown modest efficacy in randomised clinical trials. In a trial of intravenous iloprost versus aspirin [20], healing of ulcers was higher in the iloprost arm and was observed in 18 out of 52 patients receiving iloprost (35%). In another trial, an oral formulation of iloprost was not better than placebo with regard to this outcome [21]. In the aggregate, the efficacy results shown by prostacyclin analogues when used for the management of TAO are far from satisfactory. On the contrary, in our study, 10 out of 12 ulcers have improved (83%), of which eight have healed. Although these results are from a small study and are not comparable with those from randomised trials, they seem to be promising.

Giving up smoking, the only therapeutic procedure that has proven to be successful in TAO, was achieved in only four out of 12 patients (33%). This unsatisfactory rate is in accordance with previous reports which highlight the fact that it was extremely difficult for patients who are heavy smokers to give up smoking despite having strongly been advised to do so, as well as having received full information about the benefits of giving up smoking, especially in terms of avoiding amputations [22]. Patients reported their tobacco consumption and no medical diagnostic test was used to confirm the absolute cessation of smoking, which represents a limitation of this clinical study.

The majority of our patients had been treated with prostanoids, bosentan being the frontline pharmacological regimen in only two patients. Moreover, four out of the 13 target extremities had been surgically treated (lumbar sympathectomy or by-pass procedures). Thus, almost all patients included in this study should be considered refractory to previous therapeutic strategies. Taken together, the data on previous treatments as well as the high rate of smoking continuation reveal that the patients in our study were difficult to manage, given that they presented previous negative prognostic factors.

A possible explanation for the bosentan pharmacodynamic effect on endothelial function is based on the endothelial function impairment observed in patients with peripheral arterial disease in general [11] and in TAO patients in particular [23]. In addition, bosentan has shown that it can improve endothelial function, at least in patients with systemic sclerosis [24]. Thus, there is a mechanistic background to explain our results on endothelial function, which was indirectly measured in our study by means of BAFMD. In fact, BAFMD results clearly improved in every patient treated and also in the overall mean value.

Moreover, an elevated serum ET-1 level has been observed in patients with TAO, supporting a possible mechanistic explanation of the clinical benefit of bosentan in these patients [25]. Adverse events of bosentan treatment were as expected. Notably, liver tolerance was excellent and anaemia required iron supplementation in only one patient. Compliance with the treatment was also excellent (monitored by pill counts in every follow-up visit) with no patients needing to withdraw from the treatment as a consequence of adverse events.

## Conclusions

The hypothesis that bosentan treatment in TAO patients results in an improvement of clinical, angiographic and endothelial function outcomes is supported by the results of this small pilot study. Bosentan should be investigated further with regard to TAO patient management. New clinical trials with a control group should be performed to confirm our results. In the meantime, bosentan should be considered as a promising investigational agent for treating these patients.

## List of abbreviations

TAO: Thromboangiitis obliterans; ET-1: Endothelin-1; ABI: ankle-brachial index; DBI: digital-brachial index; RBI: radial-brachial index; UBI: ulnar-brachial index; BAFMD: brachial artery flow-mediated dilation test; MRI: angio-magnetic resonance imaging; NO: Nitric Oxide.

## Competing interests

The authors declare that they have no conflict of interests regarding the issues related to this study.

## Authors' contributions

All authors have participated in creating the work, and the final version of the manuscript was seen and approved by all authors. We accept full responsibility for the design and conduct of the study, and had full access to the data and controlled the decision to publish them. All co-authors agree with this and have participated in the study to a sufficient extent to be named as authors. All authors read and approved the final manuscript.

## Pre-publication history

The pre-publication history for this paper can be accessed here:

http://www.biomedcentral.com/1471-2261/12/5/prepub

## References

[B1] Olin J Young J Graor R Ruschhaupt W Bartholomew J The changing clinical spectrum of thromboangiitis obliterans (Buerger's disease) Circulation 1990 82 5 Suppl 4 3 8 2225420

[B2] Yorukoglu Y Ilgit E Zengin M Nazliel K Salman E Yucel E Thromboangiitis obliterans (Buerger's disease in women-a reevaluation) Angiology 1993 44 527 532 10.1177/000331979304400704 8328680

[B3] Yoshimuta T Akutsu K Okajima T Tamori Y Kubota Y Takeshita S Corkscrew collaterals in Buerger's disease Can J Cardiol 2009 25 365 10.1016/S0828-282X(09)70099-6 19536378 PMC2722480

[B4] Cooper LT Henderson SS Ballman KV Offord KP Tse TS Holmes DR Hurt RD A prospective, case-control study of tobacco dependence in thromboangiitis obliterans (Buerger's Disease) Angiology 2006 57 73 78 10.1177/000331970605700110 16444459

[B5] Watts SW Endothelin receptors: what's new and what do we need to know? Am J Physiol Regul Integr Comp Physiol 2010 298 R254 260 10.1152/ajpregu.00584.2009 19907001 PMC2828166

[B6] Launay D Diot E Pasquier E Mouthon L Boullanger N Fain O Jego P Carpentier P Hatron PY Hachulla E Bosentan for treatment of active digital ulcers in patients with systemic sclerosis Presse Med 2006 35 587 592 10.1016/S0755-4982(06)74645-0 16614599

[B7] Funauchi M Kishimoto K Shimazu H Nagare Y Hino S Yano T Kinoshita K Effects of bosentan on the skin lesions: an observational study from a single center in Japan Rheumatol Int 2009 29 769 775 10.1007/s00296-008-0789-z 19037604

[B8] Riccardi MT Chialà A lannone F Grattagliano V Covelli M Lapadula G Treatment of digital ulcers in systemic sclerosis with endothelin-1 receptor antagonist (bosentan) Reumatismo 2007 59 135 139 17603693 10.4081/reumatismo.2007.135

[B9] Tsifetaki N Botzoris V Alamanos Y Argyriou E Zioga A Drosos AA Bosentan for Digital Ulcers in Patients with Systemic Sclerosis: A Prospective 3-year Follow up Study J Rheumatol 2009 36 1550 1551 10.3899/jrheum.080992 19567637

[B10] Shionoya S Diagnostic criteria of Buerger's disease Int J Cardiol 1998 66 Suppl 1 S243 5 9951826 10.1016/s0167-5273(98)00175-2

[B11] Zimmerman NB Occlusive vascular disorders of the upper extremity Hand Clin 1993 9 1 139 50 8444972

[B12] Ouriel K Noninvasive diagnosis of upper extremity vascular disease Semin Vasc Surg 1998 11 2 54 9 9671234

[B13] Ruch D Koman LA Smith TL Chronic vascular disorders of the upper extremity J Am Society Surg Hand 2001 1 1 73 80 10.1053/jssh.2001.21780

[B14] Maldonado FJ Miralles J de H Aguilar EM Gonzalez AF García JR García FA Relationship between noninvasively measured endothelial function and peripheral arterial disease Angiology 2009 60 6 725 731 10.1177/0003319708327787 19054793

[B15] Norgren L Hiatt WR Dormandy JA Nehler MR Harris KA Fowkes FG TASC II Working Group. Inter-Society Consensus for the Management of Peripheral Arterial Disease (TASC II) J Vasc Surg 2007 45 Suppl S S5 67 17223489 10.1016/j.jvs.2006.12.037

[B16] Ohta T Ishioashi H Hosaka M Sugimoto I Clinical and social consequences of Buerger Disease J Vasc Surg 2004 39 176 180 10.1016/j.jvs.2003.08.006 14718836

[B17] Puéchal X Fiessinger JN Thromboangiitis obliterans or Buerger's disease:challenges for the rheumatologist Rheumatology 2007 46 192 199 17116654 10.1093/rheumatology/kel388

[B18] Todoli JA Hernández MM Arrébola MA Efficacy of bosentan in digital ischemic ulcers Ann Vasc Surg 2010 24 5 690.e1 4 10.1016/j.avsg.2010.03.011 20579585

[B19] De Haro J Florez A Fernandez JL Acin F Treatment of Buerger disease (thromboangiitis obliterans) with bosentan: a case report BMJ Case Reports 2009 doi:10.1136/bcr.08.2008.0691. Published online: May 12th 2009. [http://casereports.bmj.com/content/2009/bcr.08.2008.0691] 10.1136/bcr.08.2008.0691 PMC3029416 21686681

[B20] Fiessinger JN Schäfer M Trial of iloprost versus aspirin treatment for critical limb ischaemia of thromboangiitis obliterans. The TAO Study Lancet 1990 335 555 557 10.1016/0140-6736(90)90346-7 1689791

[B21] The European TAO Study Group Oral iloprost in the treatment of thromboangiitis obliterans (Buerger's disease): A double-blind, randomized, placebo-controlled trial Eur J Vasc Endovasc Surg 1998 15 300 307 9610341 10.1016/s1078-5884(98)80032-4

[B22] Szuba A Cooke JR Thromboangiitis Obliterans. An Update on Buerger's Disease West J Med 1998 168 255 260 9584663 PMC1304949

[B23] Makita S Nakamura M Murakami H Komoda K Kawazoe K Hiramori K Impaired endothelium-dependent vasorelaxation in peripheral vasculature of patients with thromboangiitis obliterans (Buerger's disease) Circulation 1996 94 9 Suppl 2 211 215 8901748

[B24] Sfikakis PP Papamichael C Stamatelopoulos KS Tousoulis D Fragiadaki KG Katsichti P Stefanadis C Mavrikakis M Improvement of vascular endothelial function using the oral endothelin receptor antagonist bosentan in patients with systemic sclerosis Arthritis Rheum 2007 56 1985 1993 10.1002/art.22634 17530638

[B25] Czarnacki M Gacka M Adamiec R A role of endothelin 1 in the pathogenesis of thromboangiitis obliterans (initital news) Przegl Lek 2004 61 12 1346 50 15850327

